# The Benefit of Maternal Care and Infanticide by Males as Reproductive Strategies in a Caprellid Amphipod

**DOI:** 10.1002/ece3.73364

**Published:** 2026-04-05

**Authors:** Kosei Nakano, Masakazu Aoki

**Affiliations:** ^1^ Graduate School of Agricultural Science Tohoku University Sendai Miyagi Japan

**Keywords:** Amphipoda, antagonistic behavior, cannibalism, crustaceans, parental investment, sexual selection

## Abstract

The survival of juveniles is essential for the continuation of parental genes. Consequently, the presence or potential presence of juveniles can influence the behavior of parents as well as other conspecifics. Maternal care is a key behavioral adaptation that enhances offspring survival through various mechanisms, including protection against potential threats such as infanticide. This study investigated the ability of adults to recognize juveniles and the behavioral changes induced by their presence in the marine crustacean *Caprella kominatoensis,* a species in which females are well known to provide maternal care. The role of body size in both sexes was examined to evaluate whether maternal care functions as an effective defense against male‐inflicted infanticide. It was found that females continued maternal care even after offspring were exchanged with other females, suggesting that maternal care is provided to any conspecific juveniles. When female parents were deprived of any juveniles, the period until female molting was shortened. Infanticide by males was observed only when juveniles were accompanied by the female parent; males did not harm juveniles in the absence of the female parent. Juvenile survival was higher when the female parent associated with the juveniles was larger than the male. These findings suggest that, in this caprellid amphipod, where female parents reproduce only during a brief period after molting, and molting is suppressed during maternal care, males may adopt a reproductive strategy in which infanticide facilitates the termination of maternal care, thereby accelerating female molting and increasing mating opportunities. It is concluded that maternal care is a mechanism of defense against conspecific infanticide, with female parental body size being a key factor in its effectiveness.

## Graphical abstract

In the marine amphipod Caprella kominatoensis, males perform infanticide to terminate maternal care and accelerate female molting for mating opportunities. However, we found that maternal defense is effective when the female parent is larger than the intruding male. This study highlights how sexual conflict drives the interplay between infanticide and parental care.

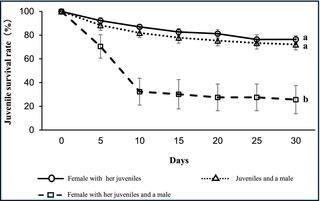

## 1 Introduction

Adult behavior and physiological state change at certain periods of the life cycle, including around the time of the hatching of juveniles (Elwood 1994). For example, adults of the amphipod *Gammarus pulex* Linnaeus, 1758, prey on nearby juveniles to supplement their nutrition. Around the time of hatching, females show reduced incidence of cannibalistic behavior (Lewis et al. 2010). In the giant water bug *Abedus breviceps* Stål, 1862 (Insecta, Order Hemiptera, Family Belostomatidae), the male carries eggs on the dorsum and shows behavioral patterns that promote oxygen flow to the eggs, with the intensity of parental care increasing as hatching approaches (Munguía‐Steyer et al. 2008). Parental investment in juveniles is of advantage to adult fitness by increasing the number and quality of juveniles able to reproduce successfully, but it also imposes costs on the reproductive capacity of the parents (Trivers 1972; Clutton‐Brock 1991). For instance, male *Abedus ovatus* Stål, 1862 (Belostomatidae) have been shown to deplete their energy reserves through parental care, potentially affecting future reproduction, in exchange for providing optimal conditions for the eggs (Argaez and Munguía‐Steyer 2024). Thus, parental care inevitably involves a trade‐off between the benefits of increasing the survival of current offspring and the costs to the future reproductive potential and survival of the parent. The presence of juveniles can affect not only the behavior of individuals performing parental care but also that of other individuals. One such behavior is infanticide, defined as the killing of immature conspecific individuals by adults of the same species. Among mammals, infanticide as an adaptive male reproductive strategy (i.e., sexually selected infanticide) was first proposed based on observations in a primate, the Hanuman langur *Semnopithecus entellus* Dufresne, 1797 (Sugiyama 1965). Langurs form social groups, and female parents rear the infants. Males from other groups invade these social groups, displace the resident males, and kill the existing infants. This is because females do not become receptive to mating while caring for their infants, but their loss alters female behavior, inducing earlier estrus. Following this report, the meaning of infanticide has been re‐examined and debated (see reviews by Hrdy 1979, Ebensperger 1998).

In marine crustaceans, maternal care is particularly well developed in the Peracarida (Johnson et al. 2001; Thiel 2003; Palaoro and Thiel 2020). Species in this group inhabit diverse environments, including freshwater, marine, and terrestrial habitats. Females are receptive to mating only for a short period immediately after molting. Consequently, when a male encounters a mature female, he performs pre‐copulatory mate guarding until she molts (Takeshita et al. 2011; Cothran et al. 2015). After assuming a copulatory posture, the male vibrates his abdomen to release sperm around the female's genital pores (Krishnan and John 1974). In this group, females lay eggs in a nursery sac, called a brood pouch, where fertilization occurs. The female then manipulates the oostegites (which form the brood pouch) through flexion movements to regulate the internal environment of the brood pouch (Dick et al. 1998). They also retrieve eggs that have fallen out (Borowsky 1980). These behaviors represent clear forms of maternal care, which have been reported to increase juvenile survival rates. However, infanticide has not been investigated in this taxon.

Caprellid amphipods are small peracarid marine crustaceans inhabiting a wide variety of substrates, including seaweeds, seagrasses, hydroids, bryozoans, artificial structures, and sandy substrates (e.g., McCain 1968; Arimoto 1976; Aoki 1999). They feed on detritus, algae, and small crustaceans (Caine 1974, 1977; Guerra‐García and de Tierno Figueroa 2009), and are themselves preyed upon by fish (Caine 1989, 1991; Edgar and Aoki 1993). Thus, they play an important role in coastal ecosystems by participating in the transfer of energy from primary producers to higher trophic levels.

Caprellids exhibit distinct sexual dimorphism (see Arimoto 1976), and morphological variation can occur even among conspecifics of the same sex, depending on the locality (Bynum 1978, 1980; Aoki and Kikuchi 1990). Their lifespan is two to three months (Takeuchi and Hirano 1991), and reproduction occurs year‐round (Caine 1979; Imada and Kikuchi 1984; Takeuchi et al. 1990). Mature females mate shortly after molting and lay eggs in a brood pouch, where embryonic development proceeds directly, and juveniles resembling miniature adults emerge.

In several caprellid species, females have been reported to engage in maternal care after juveniles leave the brood pouch, not only by feeding them but also by protecting them (Aoki and Kikuchi 1991; Thiel 1997). Aoki (1999) observed that females provide their own bodies as a substrate for the juveniles, since newly hatched juveniles are too small to effectively grasp the substrate. Maternal care in caprellids has been classified into two behavioral patterns: “clinging to mother”, in which the juveniles spend a certain period of time after birth clinging to the body of the female parent; and “stay around mother”, in which the juveniles remain in the vicinity of the female parent to be cared for (Harrison 1940; Aoki and Kikuchi 1991). Depending on the species, either or both types of care have been observed (Aoki 1997). Female caprellids provide their own bodies as substrates for juveniles that cannot grasp external surfaces, carry juveniles when escaping from predators, and engage in grooming (Aoki and Kikuchi 1991). Aoki (1997) demonstrated that, in *Caprella monoceros Mayer, 1890*, maternal care increased juvenile survival rate.

Mature females copulate during only a brief period after molting, so males adopt a strategy to secure a mating partner by precopulatory mate guarding in which they grasp a mature female using two or three pairs of their fifth to seventh pereopods until mating is possible (Lewbel 1978; Lim and Alexander 1986; Caine 1991; Aoki 1996; Takeshita and Henmi 2010). This precopulatory mate guarding is influenced by the operational sex ratio (OSR), defined as the ratio of mature males to females within a population, and studies have shown that a larger body size confers an advantage in male–male conflicts over guarding females (Takeshita and Henmi 2010). For females, precopulatory mate guarding entails costs such as reduced feeding opportunities and increased predation risk; hence, females resist guarding to shorten this period of capture by a male (Takeshita et al. 2011). Matthews (2008) confirmed that female *Caprella mutica* Schurin, 1935, displays aggressive behavior toward conspecific males, which was suggested to protect juveniles from negative impacts such as male‐inflicted infanticide. However, no studies on the function of this antagonistic behavior have been conducted to test that hypothesis.

In *Caprella kominatoensis* Takeuchi 1986, preliminary experiments discovered that female parents engaged in maternal care and repelled conspecific males. This suggests that males may impose negative effects on juveniles in this species as well, which would explain the observed female aggregation toward males to protect the juveniles. In *C. monoceros* , removing juveniles from their female parents shortens the period until molting (Aoki and Kikuchi 1991). Based on these observations, we hypothesize that *C. kominatoensis* females should show a similar pattern of maternal care and aggressive behavior toward males, and that males might commit infanticide to remove the aggressive behavior of the female (since she would have no juveniles requiring protection), thereby facilitating mate guarding until the female molts and a secure mating opportunity is secured.

In the present study, laboratory experiments were conducted using *C. kominatoensis* to (1) observe maternal care; (2) examine the occurrence of infanticide by males; (3) observe any changes in female behavior depending on the presence or absence of juveniles; and (4) test whether maternal care serves as an effective defense against male‐inflicted infanticide. These experiments included manipulation of the relative body sizes of females and males.

## 2 Materials and Methods

### 2.1 Sampling

All specimens used in the experiments were collected from the subtidal zone at Kitsunezaki, Ishinomaki City, Miyagi Prefecture. Sampling was conducted on August 30, 2022, December 13, 2022, and May 28, 2024. The substrate in the subtidal zone at Kitsunezaki is rocky and covered prominently with the rhodophyte *Gelidium elegans* Kützing distributed at a depth of 2–3 m. Specimens of *C. kominatoensis* were collected from *G. elegans* at a depth of 2–3 m by SCUBA diving.

To detach the epibenthic fauna without causing physical damage, the thalli of *G. elegans* were cut at the base and gently suspended in the water column until detachment of the epibenthic fauna. From the detached fauna, *C. kominatoensis* individuals were sorted under water and collected into 50 mL conical tubes, then transported ashore. This method was expected to minimize stress on the animals. Collected specimens were placed in 300 mL wide‐mouth bottles filled with seawater and transported back to the laboratory in a cooler box containing a few cool packs to maintain a temperature close to the ambient seawater temperature.

### 2.2 Laboratory Rearing Conditions

The captured specimens of *C. kominatoensis* were sorted into adult females, adult males, and juveniles. This was performed with reference to the original description of the species (Takeuchi 1986), which exhibits distinct sexual dimorphism. Adult females were identified by the presence of a brood pouch (oostegites) on pereonites III and IV. Even in young females, early‐stage oostegites appearing as small buds were observed. Adult males were identified by the absence of oostegites and the presence of prominently enlarged second gnathopods compared to females, or by the presence of a first antenna that was clearly longer than half the body length (typically reaching up to three‐fourths of the body length in large males). Individuals who did not exhibit these specific sexual characteristics were classified as juveniles. Only adult females and adult males were used in the following experiments.

Collected specimens were housed in separate rearing chambers (265 × 155 × 180 mm) by sex. The rearing chamber was filled with autoclaved artificial seawater (LIVESea Salt, Delphis, Itami, Hyogo, Japan) with a salinity of 32. Nylon nets (mesh approximately 1 mm) were used as substrate. The natural substrate (rhodophyte *G. elegans* ) was not used to ensure optimal water quality. Artificial feed (Tetrafin, Tetra; Blacksburg, VA, USA) was provided ad libitum, and feed from the previous day was removed the next afternoon. The chambers were placed in an incubator (EYELA FLI‐2000; Rikakikai Co. Ltd., Tokyo) set at 15°C and light/dark conditions of 12 L:12D.

Ovigerous females collected from the field were maintained individually in experimental chambers until the juveniles emerged from the brood pouch. *Caprella kominatoensis* exhibits the two typical caprellid phases of maternal care (Aoki and Kikuchi 1991; Aoki 1997): an initial “clinging” phase (2–3 days) where juveniles cling to the mother's body, followed by a “staying around” phase (20–25 days) where they aggregate near the mother (unpublished data for this species).

We defined the day of juvenile emergence from the brood pouch as ‘Day 0’. Consequently, the starting point of the experiment differed depending on the experimental manipulation required: For Experiment 1, which required the separation and exchange of juveniles between females, experiments were begun at Day 2–3 (immediately after the juveniles transitioned to the “staying around” phase). This timing was chosen to avoid physical damage or stress to the juveniles caused by forcibly detaching them during the clinging phase. For Experiments 2, 3, and 4, which did not require the separation of juveniles from their mothers, experiments were initiated at Day 0 (immediately after the juveniles emerged from the brood pouch). The number of juveniles in each replicate was recorded at the start of these experiments (Day 0) to assess survival rates.

In experiments involving males (Experiments 2 and 4), adult males collected from the field were randomly assigned to the experimental chambers. In each replicate, only one male was introduced into the chamber to avoid male–male aggression. chamber.

### 2.3 Experimental Methods

#### 2.3.1 Specimens Collected and Experiments to Which They Were Assigned

Table 1 provides an overview of each experiment. The specimens used in Experiments 1, 2, and 3 were collected on August 30 and December 13, 2022, and the experiments were conducted from September 2022 to January 2023. The specimens used in Experiment 4 were collected on May 28, 2024, and the experiment was conducted from June to August 2024. The experimental chamber (35 × 55 × 85 mm) was filled with 100 mL of autoclaved artificial seawater. As with the rearing chamber, nylon mesh was used for the substrate. During the experimental period, half of the sterile seawater was replaced daily, and artificial feed (Tetrafin, Tetra; Blacksburg, VA, USA) was provided ad libitum (all feed from the previous day was removed the next afternoon). The chambers were cleaned every 5 to 7 days. Each specimen was used for only one experimental trial (i.e., no individual was reused).

**TABLE 1:** Overview of the experimental design, including conditions, duration, and tested hypotheses for each experiment.

Experiment no.	Conditions	Duration in days	Tested hypothesis
1	Female with juveniles	(30 min)	The juvenile recognition ability in females
2	Female with her juveniles	30	Whether males perform infanticide
	Female with her juveniles and a male		
	Female removed her juveniles and a male		
3	Female with her juveniles and a male	30	Effects of juveniles' presence on the duration until the next brooding of the mother
	Female removed her juveniles and a male		
4	Female with her juveniles	30	The relation of infanticide with maternal care
	Juveniles only		
	Female with juveniles and small male		
	Female with juveniles and large male		

#### 2.3.2 Experiment 1: Identification of Offspring by the Mother

This experiment investigated whether mothers recognize their own juveniles by exchanging juveniles between females providing maternal care (*n* = 5). Experiments were initiated 2–3 days after juvenile emergence, when the maternal care behavior shifted from the “clinging” phase to the “staying around” phase.

To minimize physical damage to the fragile juveniles during handling, we exchanged the female parents between chambers instead of transferring the juveniles. Specifically, a female was removed from her original chamber (containing her own juveniles) and introduced into another chamber containing an unrelated clutch of juveniles (at the same developmental stage). Thus, each chamber contained one female and a brood of non‐filial juveniles (*n* = 5).

The behavior of the females toward the non‐filial juveniles was observed. To elicit maternal defense behaviors, an adult male was introduced into each chamber. Behavioral observations were conducted by direct visual inspection for 30 min. After the observation period, the male was removed.

The following behaviors were recorded and classified (these categories were also used in Experiments 2 and 4):

Antagonistic behavior: repelling an approaching conspecific by using the second gnathopods or by swinging the body back and forth.

Grooming behavior: cleaning the body surface of juveniles with the mouthparts.

Female escape behavior: in response to disturbance, placing nearby juveniles onto the dorsal body surface using the first antennae and then moving away from the intrusion.

Infanticide: predation on living juveniles using only the mouthparts.

#### 2.3.3 Experiment 2: Effects of the Presence and Behavior of Males on the Survival Rate of Juveniles

To investigate whether or not the presence or behavior of males affected the survival of juveniles, the following three conditions were established. Five independent replicates were conducted for each condition (i.e., *n* = 5). Each replicate used one adult male and/or one female with her natural brood. Experiments were initiated on the day of juvenile emergence (Day 0). In this experiment, specific body sizes were not measured.

- Female with her juveniles.
- Female with her juveniles and a male.
- Juveniles and a male.

All groups were maintained for 30 days. The number of juveniles in each replicate was recorded at the start (29.5 ± 5.8, mean ± SD) to assess survival rates. To calculate survival rates, the number of surviving juveniles was counted every 5 days, in the afternoon (between 13:00 and 16:00). On each of these sampling days, behavioral observations were also conducted for 10 min.

#### 2.3.4 Experiment 3: Effects of the Presence of Juveniles on the Interval Until the Next Brooding by the Mother

The following conditions were established, with independent replicates (*n* = 5) for each.

- Female with her juveniles and a male (measured simultaneously in Experiment 2b).
- Female (without her juveniles) and a male.

All groups were maintained for 30 days. The number of days until the next oviposition by the female was observed under these two conditions.

#### 2.3.5 Experiment 4: Effects of Relative Body Size of Males and Females on Juvenile Survival

To investigate any effects of the relative body size of males and females on the incidence of infanticide, the following conditions were established (*n* = 5 for each).

- Female with her juveniles.
- Juveniles only.
- Female with her juveniles and a smaller male (in comparison with the female).
- Female with her juveniles and a larger male.

All groups were maintained for 30 days, and the number of surviving juveniles was recorded every 5 days to calculate survival rates. The mean survival rates of juveniles were then compared. For conditions (c) and (d), females and males were initially classified as larger or smaller relative to each other by visual inspection. After the experiment, body sizes were measured using the image analysis software ImageJ ver. 1.54 (Schneider et al. 2012). The female‐to‐male body size ratio was 1.10 ± 0.0067 (mean ± SE) in condition (c) and 0.82 ± 0.054 in condition (d), confirming a difference of approximately 10% or more in both cases. The number of juveniles in each replicate was recorded at the start (21.7 ± 5.4, mean ± SD). In addition, for conditions (c) and (d), 30‐min video recordings (iPhone 13; Apple Inc., Cupertino, CA, USA) were made daily in the afternoon (between 13:00 and 16:00) to observe behavior.

### 2.4 Statistical Analysis

To evaluate the effects of experimental conditions on juvenile survival, a Generalized Linear Model (GLM) analysis was performed assuming a binomial error distribution and a logit link function. The response variable was defined as a two‐column matrix consisting of the number of surviving and dead juveniles in each replicate at the end of the experiment (Day 30), while the experimental condition was included as an explanatory variable. The overall significance of the model was assessed using a Likelihood Ratio Test (LRT). Subsequently, post hoc multiple comparisons were conducted using Tukey's Honest Significant Difference (HSD) test to identify specific differences among conditions. All statistical analyses were performed using R version 4.5.0 (R Core Team 2025).

## 3 Results

### 3.1 Experiment 1: Identification of Offspring by the Mother

Female parents provided maternal care even toward juveniles originating from other females (Table S4). Specifically, antagonistic behavior against males, grooming behavior, and escape behavior were observed in all examined females (*n* = 5). Grooming behavior was observed only during the early phase (Days 1–10). In contrast, antagonistic and escape behaviors persisted throughout the observation period (up to Day 30).

### 3.2 Experiment 2: Effects of the Presence and Behavior of a Male on the Survival Rate of Juveniles

**FIGURE 1:**
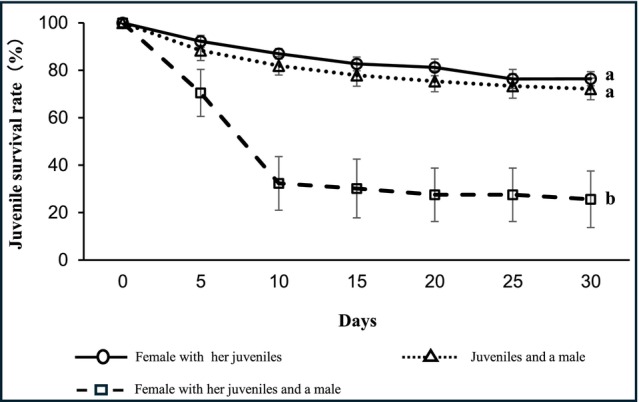
Changes in mean juvenile survival rate over 30 days in Experiment 2 (*n* = 5). Bars indicate standard error. Different lowercase letters indicate significant differences between groups (GLM with binomial error distribution followed by Tukey's HSD test, *p* < 0.001).

Females were observed providing typical maternal care behavior toward their juveniles (Table S4). According to a GLM analysis followed by Tukey's HSD test, the juvenile survival rate in the group containing a female, juveniles, and a male was significantly lower than in all other experimental groups (Table. S1; Figure 1; *p* < 0.001). In this group, males were directly observed preying on juveniles (i.e., infanticide) in two cases. Furthermore, no carcasses of the missing juveniles were found, strongly suggesting that they were consumed by the males.

In contrast, the survival rate in the group containing juveniles and a male (in the absence of a female) did not differ significantly from the other high‐survival groups.

### 3.3 Experiment 3: Effects of the Presence of Juveniles on the Interval Until the Next Brooding by the Mother

When a female was kept with the juveniles and a male, only one of five females was observed to brood a new clutch (at Day 18), while the other four did not. However, in the absence of juveniles, the females spawned eggs after 8.0 ± 0.7 days (Table S2).

### 3.4 Experiment 4: Effects of Relative Body Size of Males and Females on Juvenile Survival

Females provided maternal care behavior toward their juveniles (Table S4). However, consistent with Experiment 2, infanticide by males and antagonistic behavior by females were observed regardless of the male's size. Specifically, antagonistic behavior (1–4 times/observation) and escape behavior were recorded in all replicates for both smaller and larger male groups.

**FIGURE 2:**
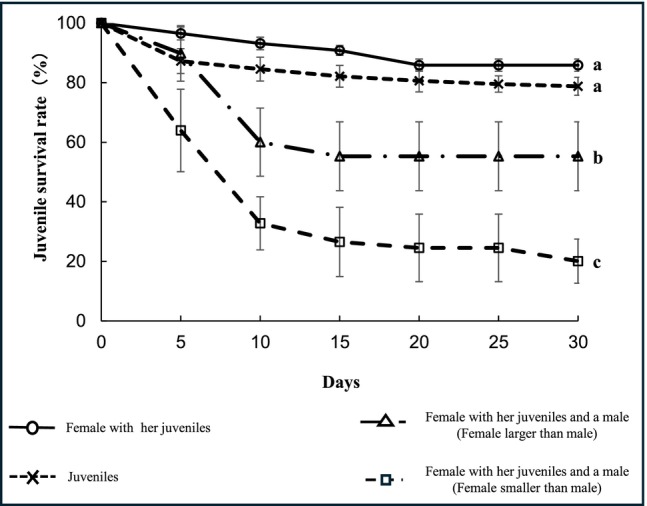
Changes in mean juvenile survival rate over 30 days in Experiment 4 (*n* = 5). Bars indicate standard error. Different lowercase letters indicate significant differences between groups (GLM with binomial error distribution followed by Tukey's HSD test, *p* < 0.001).

A GLM analysis followed by Tukey's HSD test revealed that juvenile survival rates in the presence of both smaller and larger males were significantly lower than those in the ‘juveniles only’ and ‘female with juveniles’ groups (Table S3; Figure 2; *p* < 0.001). Furthermore, the survival rate in the presence of a larger male was significantly lower than that in the presence of a smaller male (*p* < 0.001).

## 4 Discussion

In the present study, male predation on juveniles was observed in C. kominatoensis under conditions where a female, juveniles, and a male were kept together, and juvenile survival rates were lower than in the other experimental groups (Figures 1 and 2). In contrast, when juveniles were kept with a male only, their survival rate was comparable to that of the ‘female with juveniles’ group (Figure 1). The removal of juveniles from the mother resulted in a shorter period until the female molted. When juveniles were exchanged between female parents, the female parents continued maternal care. In addition, when the body size of mothers exceeded that of an accompanying male, the decline in juvenile survival was less pronounced (Figure 2), indicating that parental care may function as a defensive strategy against infanticide by males.

Previous reviews of parental care (Palaoro and Thiel 2020) and cannibalism in marine crustaceans (Bleakley et al. 2018) did not mention infanticide. Therefore, the observation of infanticide by male *C. kominatoensis* reported in the present study appears to represent the first record of such behavior in marine crustaceans. In contrast, infanticide has been widely studied in terrestrial mammals, and five major hypotheses have been proposed to explain its occurrence (see reviews by Hrdy 1979; Ebensperger 1998), which can be considered in trying to explain the occurrence of infanticide observed in *C. kominatoensis*. First, the predation hypothesis proposes that infanticidal individuals consume juveniles to obtain nutritional benefits. In the presence of females, juvenile survival was reduced due to observed infanticide, whereas survival rates remained high when juveniles were kept with a male alone (Figure 2). This suggests that males do not inherently view juveniles as a food source, leading us to reject the predation hypothesis.

Second, the resource competition hypothesis posits that infanticide eliminates potential competitors for limited resources. However, Experiment 2 was conducted under food‐satiated conditions, and males did not eliminate juveniles in the absence of the female. The observation that infanticide occurred only when males were in the presence of the female strongly suggests that juveniles were not targeted as competitors for food or space, rendering this hypothesis unlikely.

Third, the adoption avoidance hypothesis suggests that individuals commit infanticide to avoid investing in unrelated offspring. Our observations in Experiment 1 confirmed that all five females continued to perform specific maternal behaviors, including grooming and antagonistic defense against males, toward the unrelated juveniles (Table S4). Although fine‐scale behavioral frequencies were not compared, the consistent occurrence of these active care behaviors in all replicates implies that females do not discriminate against unrelated juveniles, or that the “maternal state” persists strongly enough to override kin recognition.

Fourth, the non‐adaptive hypothesis attributes infanticide to social pathology caused by stress. While laboratory conditions can induce stress, the fact that males did not harm juveniles in the absence of females (Figure 1) but specifically targeted them in the presence of females suggests that the behavior is not merely a maladaptive response, but a context‐dependent social interaction.

Finally, the sexual selection hypothesis argues that individuals kill juveniles to terminate parental care and accelerate the mating partner's return to sexual receptivity. Our results in Experiment 3 demonstrated that the removal of juveniles significantly shortened the time until the female molted and spawned eggs compared to females that retained their juveniles (Table S1). This finding confirms the physiological mechanism required for the sexual selection hypothesis. Therefore, among the five hypotheses, the sexual selection hypothesis provides the most plausible explanation for infanticide in this species.

Although the sexual selection hypothesis is the most plausible, the male's success rate appears variable. In Experiment 2, where males committed infanticide, only one of five females brooded a new clutch within the observation period. This discrepancy suggests that while infanticide is a strategy aimed at inducing mating, it is not always immediately successful due to female resistance. For the “maternal state” to end and the “mating state” to resume, it is likely that the brood must be lost completely or reduced below a critical threshold. A similar phenomenon has been reported in the giant water bug *Lethocerus deyrollei* , Vuillefroy, 1864 (Ichikawa 1990). In this species, females destroy egg masses guarded by males to gain mating opportunities, but males abandon parental care and accept new mates only when the egg mass size is reduced to a specific low number. This suggests that thorough destruction is required to reset the parental state. In present experiments, maternal defense often prevented the complete elimination of juveniles (i.e., keeping the brood size above the abandonment threshold). This partial success of infanticide highlights an intense sexual conflict: while males attempt to kill juveniles to gain mating opportunities, female defense acts as a robust counter‐strategy, effectively creating a stalemate that prevents males from easily achieving their goal.

The effectiveness of this maternal defense appears to depend on the body size ratio between the parents. Previous studies on caprellids have yielded conflicting results regarding the role of body size in male–male competition: while Lim and Alexander (1986) reported that size ratio did not determine conflict outcomes in *Caprella scaura* , Takeshita and Henmi (2010) showed that size conferred a clear advantage in *C. penantis* . In the context of inter‐sexual conflict observed in the present study, body size ratio played a critical role: although survival rates were lower in the presence of males regardless of size, the decline was significantly less pronounced when the male was smaller than the female (Figure 2). This indicates that parental care functions as an effective defensive strategy against infanticide, particularly when the female has a physical advantage over the intruder.

This study has several limitations that should be noted. First, our experiments were conducted under simplified laboratory conditions using small containers. In the wild, complex habitat structures and larger spaces would provide more refuges for juveniles and females, potentially altering the frequency of male–female encounters and the success rate of infanticide. Additionally, the absence of natural predators in the laboratory might have allowed males to engage in conspicuous behaviors such as infanticide more frequently than they would in nature, or conversely, forced females to defend their brood more aggressively due to the lack of escape routes.

Second, the survival rate of juveniles was monitored at 5‐day intervals. This sampling frequency was too coarse to capture the precise timing of infanticide (e.g., immediately after male introduction) and may have missed short‐term behavioral interactions. Future studies should employ more frequent (e.g., daily) or continuous monitoring, as well as field experiments, to clarify the detailed temporal dynamics of male behavior and juvenile mortality under natural conditions. In this study, infanticide in *C. kominatoensis* was reported for the first time. Since maternal care is known to occur in various amphipod species, including caprellids, and reproductive strategies are highly diverse, it is possible that infanticidal behavior also occurs in other groups of amphipods.

## Author Contributions

**Kosei Nakano:** conceptualization (lead), data curation (lead), formal analysis (lead), funding acquisition (lead), investigation (lead), methodology (lead), resources (equal), validation (equal), visualization (lead), writing – original draft (lead), writing – review and editing (equal). **Masakazu Aoki:** conceptualization (equal), funding acquisition (supporting), investigation (supporting), methodology (equal), project administration (lead), resources (equal), supervision (lead), validation (equal), writing – review and editing (equal).

## Funding

We gratefully acknowledge financial support for this study provided by the Research Institute of Marine Invertebrates (Grant No. IKU2023–02).

## Ethics Statement

The authors have nothing to report.

## Conflicts of Interest

The authors declare no conflicts of interest.

## Supporting information


**Table S1:** ece373364‐sup‐0001‐TableS1.xlsx.

**Table S2:** ece373364‐sup‐0002‐TableS2.xlsx.

**Table S3:** ece373364‐sup‐0003‐TableS3.xlsx.

**Table S4:** ece373364‐sup‐0004‐TableS4.xlsx.

## Data Availability

The data that supports the findings of this study are available in the Supporting Information of this article.
